# International Horizon Scanning the impact of Covid-19 on increasing the health gap and vulnerability: Liz Green

**DOI:** 10.1093/eurpub/ckac131.005

**Published:** 2022-10-25

**Authors:** A Instone, A Stielke, J Allen, A Cotter-Roberts, B Bainham, L Couzens, C Beynon, M Dyakova, L Green

**Affiliations:** 1 WHO Collaborating Center on Investment for Health and Well-being, Public Health Wales, Cardiff, UK; 2 Health Improvement, Public Health Wales, Cardiff, UK

## Abstract

The COVID-19 pandemic has caused unprecedented challenges for populations, health systems and governments worldwide, which have resulted in lasting economic, social and health impacts. The results of such have been felt disproportionately throughout society and existing vulnerabilities have been highlighted and heightened. A clear understanding of the extent of these vulnerabilities is needed in order to fully address the problem. The World Health Organization Collaborating Centre on Investment for Health and Well-being (WHOCC), Public Health Wales has developed a summary report focusing on the existing and emerging inequalities resulting from the pandemic, as identified through international evidence and learning from the International Horizon Scanning Reports. These reports, undertaken between May 2020 - August 2021, are based upon rapid evidence synthesis reviews of international literature. The summary report focuses on global learning and best practices in order to better understand and address the unequal impacts of the pandemic. The information has been categorised according to the five essential conditions required to enable a healthy life as presented within the WHO health equity conditions framework. The report provides evidence on groups most vulnerable to both direct and indirect impacts of the pandemic as well as promising practice to address the resulting inequity. Inequalities and related factors explored within the report include but are not limited to, level of deprivation and education. Taking a global perspective, this report summarises international evidence to support inclusive, sustainable, and equitable solutions, such as protecting economic well-being and taking an intergenerational lens in both response and recovery. To address and mitigate the impact of the pandemic upon vulnerable groups, collating and sharing international evidence and best practice has proven to support equitable long-term socio-economic and environmental recovery.

**Key messages:**

• International learning provides vital insights to support recovery in Wales and beyond.

• Responses to the pandemic should address the needs of the vulnerable to reduce existing health gaps.

